# Millimetre-Wave Backhaul for 5G Networks: Challenges and Solutions

**DOI:** 10.3390/s16060892

**Published:** 2016-06-16

**Authors:** Wei Feng, Yong Li, Depeng Jin, Li Su, Sheng Chen

**Affiliations:** 1 State Key Laboratory on Microwave and Digital Communications, Tsinghua National Laboratory for Information Science and Technology (TNLIST), Department of Electronic Engineering, Tsinghua University, Beijing 100084, China; fengw12@mails.tsinghua.edu.cn (W.F.); jindp@tsinghua.edu.cn (D.J.); lisu@tsinghua.edu.cn (L.S.); 2 School of Electronics and Computer Science, University of Southampton, Southampton SO17 1BJ, UK; sqc@ecs.soton.ac.uk; 3 King Abdulaziz University, Jeddah 21589, Saudi Arabia

**Keywords:** millimetre-wave backhaul, 5G, multi-hop routing, scheduling, hybrid beamforming, full duplexing

## Abstract

The trend for dense deployment in future 5G mobile communication networks makes current wired backhaul infeasible owing to the high cost. Millimetre-wave (mm-wave) communication, a promising technique with the capability of providing a multi-gigabit transmission rate, offers a flexible and cost-effective candidate for 5G backhauling. By exploiting highly directional antennas, it becomes practical to cope with explosive traffic demands and to deal with interference problems. Several advancements in physical layer technology, such as hybrid beamforming and full duplexing, bring new challenges and opportunities for mm-wave backhaul. This article introduces a design framework for 5G mm-wave backhaul, including routing, spatial reuse scheduling and physical layer techniques. The associated optimization model, open problems and potential solutions are discussed to fully exploit the throughput gain of the backhaul network. Extensive simulations are conducted to verify the potential benefits of the proposed method for the 5G mm-wave backhaul design.

## 1. Introduction

The commercial operation of 5G is expected to start globally by 2020. The new generation mobile communication is estimated to provide 1000-times more capacity and support 100-times more smart devices than current cellular networks [1]. Among all traffic demands, services requiring a high transmission rate, such as video streaming, are predicted to be the dominant service type according to the statistics obtained by Cisco. Inevitable dense deployment of mobile terminals and infrastructures makes a heterogeneous network (HetNet) a crucial solution to meet the capacity growth [2]. Cost-effective small cells are connected with macrocells in HetNet to extend coverage in crowded environments [3].

Densification of small cells produces massive backhaul traffic in the core network, which inevitably becomes an important, but somewhat less addressed bottleneck in the system. Employing fibre or cable in dense small cell backhaul would result in prohibitively high cost and practical difficulty in implementation [4]. Wireless backhaul may offer a scalable and cost-effective solution. However, traditional microwave frequency bands, e.g., sub-5 GHz, may be limited in achievable gains due to the existing spectrum crunch. Despite emerging mechanisms to enhance spectrum efficiency, it remains hard to achieve data rates exceeding 1 Gbps or even 10 Gbps. Therefore, it is difficult to meet the rapid increase of 5G traffic demands in such relatively low frequency bands. Moreover, interference becomes a limiting factor, especially in densely-populated HetNets.

Millimetre-wave (mm-wave) techniques ranging from 30 to 300 GHz have become feasible and promising means to overcome the above-mentioned issues [5]. Currently, the three most potential mm-wave bands include 28 GHz, 60 GHz and the E-band, which are mostly unlicensed. With a huge available bandwidth, the gigabit data rate is practically achievable, which solves the capacity problem that exists in lower frequency backhaul systems. In mm-wave networks, directional links are commonly established to compensate for the high path loss. The use of highly steerable antennas greatly reduces interference, and high penetration losses due to walls and other obstacles also mitigates several interfering signals. Recently, the feasibility of mm-wave transmission within a range of a few hundred meters is proven by systematic outdoor measurements [6,7], which offer sufficient coverage for small cell backhaul. Although mm-wave backhaul seems to offer a promising solution, increasing demands for 5G communication bring new challenges, which must be overcome in order to make mm-wave backhaul applicable to various scenarios. Traditional mm-wave techniques are mostly focused on direct single user link, but supporting multi-streams for multi-user scenarios is likely to be a trend for future 5G backhaul with growing traffic demands. Directional communication paves the way for spatial reuse, which should be fully exploited to improve overall backhaul performance. Moreover, high distance loss and blockage severely affect mm-wave network capacity, which requires suitable routing schemes to establish relaying links dynamically.

In this article, we establish a novel framework for 5G mm-wave backhaul based on a general and tractable system architecture. The proposed framework combines promising physical layer techniques, such as hybrid beamforming and full-duplex transmission, with routing and scheduling schemes in higher layers. Specifically, we solve the problems of transmission path selection and time allocation in mm-wave backhaul by proposing an optimization model, and we carry out the corresponding algorithmic analysis. Extensive simulations with realistic assumptions have shown that new features of physical layer techniques are capable of bringing great performance enhancement, in terms of capacity and latency in a mm-wave backhaul network. The overall objective of this article is thus to offer an overview of a design framework of mm-wave backhaul based on current research and to provide solutions of utilizing mm-wave band techniques in building a 5G backhaul network.

We structure the article as follows. After introducing the development of mm-wave techniques, we offer an overview of the mm-wave backhaul system and outline the associated challenges. Then, we introduce emerging progress in mm-wave physical layer techniques and propose an integrated design framework based on the new techniques to tackle the problems, with the demonstration of routing and scheduling based on the proposed optimization model. Finally, the capability of the proposed framework and design method is assessed via systematic simulations under a realistic scenario.

## 2. Millimetre-Wave Technology: State-of-the-Art and Trends

The mm-wave technology has long been an attractive field for both academia and industry. Numerous research literature works have investigated the features of mm-wave channels and electronics, as well as sought for ways to fully utilize these features for achieving a higher data rate. Furthermore, standardization bodies devote great efforts to establish a norm for mm-wave communication applications. For example, European Computer Manufacturers Association (ECMA)-387, IEEE 802.15.3c, IEEE 802.11ad and IEEE 802.11aj are all existing 60-GHz industrial standards, among which IEEE 802.11ad [5] is the most frequently- used one. The task group of the next generation of 60 GHz standards, IEEE 802.11ay, has been recently formed, aiming to provide at least a 20-Gbps data rate. Accordingly, mm-wave products have begun to appear in the mass market, such as the 60-GHz chips manufactured by Qualcomm.

Despite these supports, mm-wave bands have been deemed unattractive for cellular communications for a long period. The main concern is the physical barrier, including high path loss and other degradation losses, which confines mm-wave applications in an indoor environment. However, the works of New York University [6] demonstrated the feasibility of mm-wave transmission in the small cell of 4G communication under various urban environments at 28-GHz, 38-GHz and 73-GHz bands based on extensive measurement data. Similarly, the work [7] dispelled several myths on the 60 GHz band by measurements and simulations, showing the potential of mm-wave techniques in outdoor wireless networks. An overview of the above-mentioned results is summarized in Table 1. Clearly, the 28-GHz and 38-GHz bands suffer less total loss, but have a smaller bandwidth, while the 60-GHz and 73-GHz bands have larger loss, but the larger bandwidth and smaller antenna size may both contribute to compensate for this loss. Given the coverage, the effects of atmospheric and rain attenuation are small, compared to the path loss in mm-wave bands.

Encouraged by these results, mm-wave communication has recently been highlighted as a promising, enabling and disruptive technology for the future 5G system. In February 2014, Samsung announced a mm-wave beamforming prototype, with a bandwidth in excess of 500 MHz at 28 GHz. Hybrid beamforming is adopted in the prototype, which can achieve a peak rate of 1.056 Gbps and guarantees more than 500 Mbps in an area with a-few-hundred-meter radius even in a non-line of sight (NLOS) environment [8]. In August 2014, Millimetre-Wave Small Cell Access and Backhauling (MiWaveS), a European collaborative project, was been released, the goal of which is to provide a gigabit data rate for 5G wireless communication in the frequency band of 60 GHz and the E-band [9]. A baseband modem for a mobile wireless backhaul has been introduced in [10]. In the survey and design of future 5G mm-wave backhaul, the large amount of previous databases and results may help as important references.

Recently, there appeared numerical surveys and works that envisaged adopting mm-wave backhaul for 5G communications. Niu *et al.* [11] investigated joint scheduling of access and backhaul links in mm-wave small cells by considering device-to-device communications to further improve system performance. Gao *et al.* [12] proposed a promising mm-wave backhaul-based massive MIMO scheme for a 5G ultra-dense network. The proposed scheme enables multi-user MIMO with multiple streams for each user. Dehos *et al.* [13] discussed the feasibility of adopting mm-wave wireless backhaul. Two business models using E-band and 60 GHz have been compared and analysed, as well as the choices for the backhaul transceiver have been discussed in terms of performance and cost. In [14], Zheng *et al.* pointed out several MAC and networking design challenges for mm-wave small cells. Specifically, the choice of routing metrics and hierarchical schemes was discussed for designing an efficient multi-hop routing protocol in the backhaul system. However, these mm-wave backhaul-related literature works either focus on exploiting one particular technique or techniques for one specific layer or mainly introduce and discuss the challenges and requirements from a global view without proposing possible solutions. Therefore, a system-level design framework is necessary to specify the procedures of mm-wave backhauling, including how to select the transmission path and to determine the transmission sequence, *etc.*, in order to fully exploit the benefits of mm-wave techniques and to overcome the associated problems. In addition, jointly considering the new physical layer techniques with upper layer designs is necessary to enhance the overall system performance. Hence, there is still a long way to go to the final systematic implementation.

## 3. System Overview and Problem Statement

### 3.1. System Overview

Compared to traditional cellular networks, small cells in HetNets have smaller coverage, but higher capacity and less interference, all of which make mm-wave techniques naturally applicable in such scenarios. Typically, there are two options of system architecture for 5G backhaul. One is a centric solution, and the other a distributed solution [4]. In a centric solution, a macrocell base station (BS) is situated in the center, with small cell BSs (SBSs) uniformly distributed around. Direct links between SBSs are not allowed. Instead, they are required to get access to the core network through the center macrocell BS, which is connected to the gateway by fibre links. In a distributed solution, all backhaul data are relayed to a single specific wired SBS instead of the macrocell BS. Backhaul data are allowed to transmit between the established mm-wave links of adjacent SBSs and finally are collected by the designated wired SBS to the core network through fibre links. System-level simulations have shown that the distributed solution achieves higher energy efficiency and throughput gain, mainly due to sharing cooperative traffic among multiple wireless SBSs [4].

**Figure 1:**
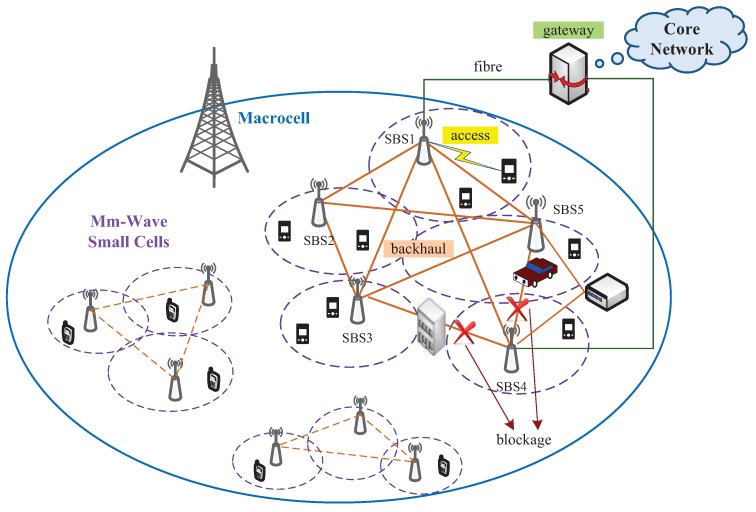
System overview for 5G mm-wave backhauling.

In this article, a more general system architecture is adopted, which is an extension of the distributed solution. An example of the system containing five small cells is illustrated in the right part of Figure 1. In this model, more than one SBSs (SBS1 and SBS4 in this example) is connected to the gateway by fibre links. As usual, backhaul data can be transmitted between SBSs through mm-wave links. Since a mm-wave link can be established between any two SBSs, a backhaul mesh network is formed by all of the SBSs. Moreover, there are two ways of backhaul data transfer: wired and wireless ways. For example, if SBS2 wants to transmit data to SBS4, it can either choose pure mm-wave links (both the direct link and relayed links are allowed) or sends the data to a wired SBS, *i.e.*, SBS1, which then forwards the data through the fibre link to SBS4. Since mm-wave communication suffers from severe penetration loss, which makes blockage a serious and common problem in backhaul networks, such a multi-way data transfer architecture makes the backhaul network more reliable. Two types of blockage are depicted in this architecture. On the one hand, the link between SBS4 and SBS5 is affected by a temporary blockage, e.g., a moving vehicle. It may require the SBSs to adopt an effective beam tracking method to switch to an alternative link after detecting link interruption and to switch back when the blockage disappears. On the other hand, the blockage between SBS3 and SBS4 is a permanent one, e.g., a building. although such situations may be mitigated to some extent by the NLOS path through reflection, they result in the largely reduced channel capacity. In this case, transmitting data to adjacent SBSs as relays may be a more suitable solution.

### 3.2. Challenges and Design Goals

Based on this backhaul system architecture, several challenges as the consequence of adopting mm-wave links and corresponding design goals for our proposed mm-wave backhaul framework are summarized as follows.

#### 3.2.1. Overcoming Path Loss

It is widely acknowledged that one of the biggest challenges of mm-wave communication is high path loss. The link attenuations at the 28 GHz and 60 GHz bands are approximately 21 dB and 28 dB larger than experienced in the conventional 2.4-GHz system with the same transmission distance, respectively. In order to compensate for such large loss, beamforming techniques are necessary in mm-wave backhauling. Benefiting from the tiny wavelength and progress in the modern complementary metal-oxide semiconductor (CMOS) process, complex antenna arrays can easily be integrated into smart devices at a low cost over a wide gigahertz frequency band [15]. Highly steerable beams are generated to form directional links. Beamforming codebooks, a group of predefined antenna array weight factors, are commonly adopted in mm-wave standards, such as IEEE 802.15.3c and IEEE 802.11ad [5]. However, with the continuous increase of data demands and service types, more efficient and adaptive beamforming techniques are required to satisfy such requirements.

#### 3.2.2. Efficient Spatial Reuse

The ultimate goal of backhaul transmission is improving throughput. Highly directional transmissions in mm-wave communication lead to much less severe interference compared to conventional wireless cellular networks, which paves the way for spatial reuse to effectively save frequency and time resources, as well as to enhance throughput gain. Spatial reuse is particularly suitable for the dense small-cell scenarios, because the densely distributed devices bring much greater opportunity for concurrent transmissions. Time division multiple access (TDMA) is adopted in the IEEE 802.11ad standard, and spatial TDMA (STDMA) has attracted significant attention recently. Transmission is conducted in units of equal-length time intervals, termed time slots. The goal of scheduling is to allocate as many links as possible in the same time slot to fully exploit spatial reuse. Currently, most mm-wave-related scheduling studies are based on the half-duplex links in the single user scenario, *i.e.*, adjacent links having a common node cannot transmit simultaneously, and a device can only either send or receive a single data stream at a time. However, recent advances in physical layer techniques, such as full duplexing, hybrid beamforming and multicast beamforming, may bring changes to the scheduling to further improve spatial reuse gain.

#### 3.2.3. Dynamic Link Establishment

In a mm-wave backhaul network, only a small number of SBSs is connected to the gateway through fibre, and backhauling relies heavily on mm-wave links. These mm-wave links can be established dynamically, which adds great flexibility to backhaul data transmission. Normally, the line of sight (LOS) path is preferred when performing wireless communication between SBSs. However, due to the high path loss and penetration loss, far-away devices and blocked links may require relays, *i.e.*, multi-hop transmissions to accomplish backhauling. In either scenario, both wireless SBSs and wired SBSs can be activated as relays. How to design an appropriate transmission route for each service becomes an important issue. Multiple factors must be taken into consideration in routing process, such as reducing the total latency, optimizing overall data flow, guaranteeing the robustness of network connectivity and providing high quality of service (QoS), *etc*.

## 4. Emerging Mm-Wave Physical Layer Techniques

**Figure 2:**
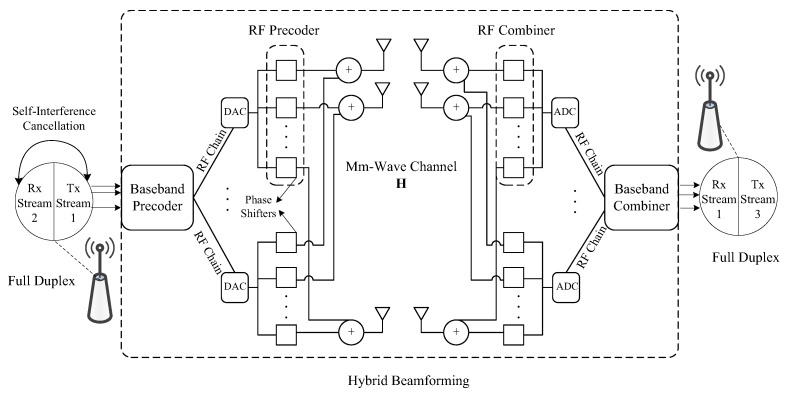
Physical layer techniques: hybrid beamforming and full duplexing.

Physical layer techniques are the most fundamental ones to satisfy data demands and basic service requirements. Mm-Wave standards, such as IEEE 802.11ad, offer various modes for different application scenarios. Due to large path loss in the mm-wave system, beamforming has been undoubtedly conceived of as a vital component for mm-wave systems. However, constraints of one radio frequency (RF) chain in each link and point-to-point transmission have made it hard for the current analogue beamforming techniques to handle the rapid increasing data demands. Beamforming techniques that support multiple RF streams become more appealing, and the emergence of a related simple prototype [8] shows the feasibility of implementation. Meanwhile, the progress in self-interference cancellation technology is likely to enable full duplexing, which helps to further improve the link capacity. Figure 2 shows an overview of these two physical layer techniques.

### 4.1. Hybrid Beamforming

Digital MIMO processing, e.g., singular value decomposition, offers great flexibility and high performance, but suffers from high complexity and power consumption in mm-wave systems. Traditional mm-wave analogue beamforming is used in antenna arrays to form highly directional beams, which has low complexity in the RF domain, but its potential is limited by single-stream transmission and the constraints of the quantized phase shifters. Therefore, by combining them together, hybrid beamforming offers a desired performance and complexity trade-off [16,17]. As can be seen from Figure 2, transmitted signals go through a digital baseband precoder, an RF analogue transmit and receive beamformer and a digital baseband combiner. Multiple RF chains allow multi-stream multiplexing between the transmitter and the receiver, which can largely enhance the overall throughput if adopted in backhaul. Hybrid beamforming is also applicable in multiuser scenarios, enabling the transmitter to send multiple streams to independent users simultaneously [18], which may further improve the backhaul network capacity by supporting concurrent multiuser transmissions.

### 4.2. Full Duplex Transceiver

Half duplexing has been a long-held assumption in wireless communication. In 2013, Bharadia *et al.* from Stanford University [19] presented a design for in-band full duplex WiFi radios, which can transmit and receive data simultaneously through a single antenna. The implementation shows that it almost doubles the throughput, and the design maintains the robustness in a noisy environment. In 2014, Rajagopal *et al.* at Samsung [20] showed that 70 to 80 dB isolation is available from the transmit sector to the adjacent receiver sector on the same SBS for a 28-GHz mm-wave prototype adopting directional antenna arrays. They also illustrate that an additional 30 to 50 dB isolation is likely to be achieved by using conventional full duplex interference cancellation. Therefore, commercial full duplex for in-band mm-wave systems will be available soon. Using full duplex in backhaul can greatly enhance the throughput, and the benefits are especially remarkable in multi-hop routes.

## 5. Framework Design for 5G Mm-Wave Backhaul

**Figure 3:**
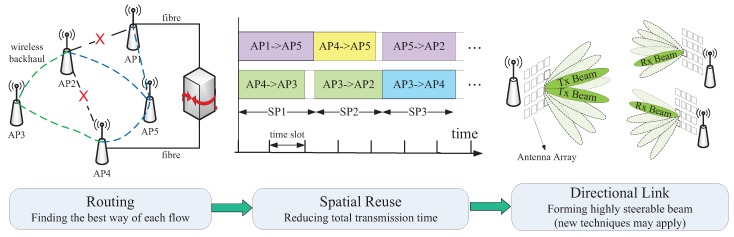
System framework for 5G mm-wave backhauling.

Aiming to achieve the desired goals in Section 3 with the support of emerging techniques in Section 4, we propose a framework for mm-wave backhaul, an overview of which is shown in Figure 3. According to the backhaul data demands, a routing scheme is needed for every flow to guarantee a robust performance in terms of latency and throughput. Then, the STDMA scheduling in the MAC layer is followed to assign specific time slots to different hops of every flow, where multiple transmissions can be allocated in the overlapping time periods to fully exploit spatial reuse gain. Finally, physical layer technologies, especially emerging high rate and reliability-aided techniques, such as hybrid beamforming and full duplex transmission, are utilized to satisfy the transmission requirements in the 5G mm-wave backhaul system.

### 5.1. MAC Layer Procedure

Currently, there is no specific industrial standard for 5G mm-wave small cells. However, existing mm-wave standards, e.g., IEEE 802.11ad, could be considered as useful references, in order to define MAC layer operations of backhaul routing and scheduling. In this paper, we borrow the idea of the cluster mechanism in IEEE 802.11ad, where several nearby small cells form a cluster. One SBS acts as the synchronization SBS (S-SBS), which provides synchronization to other SBSs, and S-SBS is able to exchange information by transmitting and receiving Beacon frames and Announce frames to and from other SBSs. After beamforming training between SBSs in a cluster, the transmitter SBS sends directional channel quality measurement request to the receiver SBS and then receives the measured signal to noise ratio (SNR) or signal to interference-plus-noise ratio (SINR) value via the directional channel quality measurement report for each backhaul link. Thus, the achievable rate for every backhaul link could be estimated and stored in a channel capacity matrix. Since the positions of SBSs are almost stationary, it is unnecessary to update the capacity matrix frequently unless a big drop occurs in system performance.

In the mm-wave backhaul system, the network time is partitioned into consecutive non-overlapping time periods, termed frames. Each frame is divided into two parts: frame header and data transmission interval. In the frame header, routing and scheduling schemes are performed for the backhaul required flows accumulated at the S-SBS in the previous frame. The S-SBS sends the result of path selection and scheduling to all other SBSs, and backhaul flows are transmitted according to this result in the data transmission interval.

In addition, Niu *et al*. [21] has proposed a software-defined cross-layer design for the mm-wave network. A logically-centralized controller, which usually resides on the gateway, is introduced to make rules and control the behaviours of SBSs globally over the system from the network layer to the physical layer. Optimized routing path selection and spatial reuse scheduling for backhaul links could be achieved through the software-defined and cross-layer controlling if the proposed architecture becomes applicable in future 5G mm-wave communications.

### 5.2. Transmission Path Selection

Multi-hop relaying is required in mm-wave backhaul to overcome blockage between SBSs or a long distance between communicating SBSs. Typically, the routing problem can be depicted by a graph, with vertices representing SBSs and edges standing for wireless or wired links. Dijkstra’s algorithm and Floyd–Warshall’s algorithm are two typical methods to search for the minimum cost path in a weighted graph. The selection of edge cost is rather flexible. For example, if we set the edge weights to one for all connected links and *∞* for blocked ones, the solution achieves the minimum number of relaying hops. The weight can also be distance for attaining the shortest-path solution and delay for obtaining the minimum-latency solution, *etc*.

Most existing wireless routing methods for mm-wave systems assume single-stream transmission. Unlike in a conventional wireless system, the routing in the proposed framework can establish a number of routes for a single flow, *i.e.*, a source may select multiple paths to relay data to the destination as a benefit of hybrid beamforming. Moreover, multiple flows can be transmitted simultaneously between two devices. Here, we introduce an optimization model for graph routing, taking physical layer techniques of hybrid beamforming and full duplex into consideration. Based on the system architecture depicted in Figure 1, we assume that there are *N* SBSs in the system denoted by $B=\{b_{1},b_{2},\ldots ,b_{N}\}$. The number of required flows are *K*, with $\left\{s_{k},d_{k}\right\}$ representing the source and destination of the *k*-th flow, respectively, for 1≤k≤K. We can then formulate the path selection in mm-wave backhaul by the following optimization problem.

Optimization objective: Network capacity is crucial for the system performance. Thus, we choose to maximize the total transmission rate as the optimization objective:

(1) $$\max\sum_{k}\sum_{b\in B,b\neq s_{k}}r_{s_{k}\to b}^{k}$$

where $r_{s_{k}\to b}^{k}$ is the transmission rate from the source to the next SBS *b* of the *k*-th flow. The objective can also be QoS [22], by choosing the cost function arguments $r_{s_{k}\to b}^{k}$ based on some appropriate QoS metrics.

System constraints: Related to the physical layer techniques in mm-wave backhauling, the constraints of the routing problem are listed as follows, which fall into two categories.

(2a) $$\begin{array}{c} r_{b_{i}\to b_{j}}^{k}\left\{\begin{array}{c} =0,\;\;\mathrm{if}\;p_{b_{i}\to b_{j}}^{k}=0\\ >0,\;\;\mathrm{if}\;p_{b_{i}\to b_{j}}^{k}=1 \end{array}\;\;\;\;\;\forall \;b_{i},b_{j}\in B,\;\forall \;k\right. \end{array}$$

(2b) $$\begin{array}{c} p_{b_{i}\to b_{j}}^{k}+p_{b_{j}\to b_{i}}^{k}\leq 1\;\;\;\;\;\forall \;b_{i},b_{j}\in B,\forall \;k \end{array}$$

(2c) $$\begin{array}{c} \sum_{k}\sum_{b_{j}}p_{b_{i}\to b_{j}}^{k}\leq f\left(N_{RF}\right)\;\;\;\;\;\forall \;b_{i}\in B,\;\;\;\sum_{k}\sum_{b_{i}}p_{b_{i}\to b_{j}}^{k}\leq f\left(N_{RF}\right)\;\;\;\;\;\forall \;b_{j}\in B \end{array}$$

(2d) $$\begin{array}{c} \sum_{b_{i}}p_{s_{k}\to b_{i}}^{k}>0,\;\sum_{b_{i}}p_{b_{i}\to d_{k}}^{k}>0\;\;\;\;\;\forall \;k \end{array}$$

(2e) $$\begin{array}{c} \sum_{b_{i}}p_{b_{i}\to s_{k}}^{k}=0,\;\sum_{b_{i}}p_{d_{k}\to b_{i}}^{k}=0\;\;\;\;\;\forall \;k \end{array}$$

(2f) $$\begin{array}{c} \sum_{b_{i}}r_{b_{i}\to b_{l}}^{k}=\sum_{b_{j}}r_{b_{l}\to b_{j}}^{k}\;\;\;\;\;\forall \;b_{l}\in B,\;b_{l}\;\neq \;s_{k},\;b_{l}\;\neq \;d_{k},\;\forall \;k \end{array}$$

(2g) $$\begin{array}{c} \sum_{k}r_{b_{i}\to b_{j}}^{k}\leq C_{b_{i}\to b_{j}}\;\;\;\;\;\forall \;b_{i},b_{j}\in B \end{array}$$

- Device and channel constraint: We define a binary variable $p_{b_{i}\to b_{j}}^{k}$ to indicate that $b_{i}$ sends data to $b_{j}$ of the *k*-th flow if it equals one, and otherwise, it is zero. The relation between $p_{b_{i}\to b_{j}}^{k}$ and transmission rate $r_{b_{i}\to b_{j}}^{k}$ is shown as Constraint (2a). Transmission cycles should be avoided in the routing, which means that $p_{b_{i}\to b_{j}}^{k}$ and $p_{b_{j}\to b_{i}}^{k}$ cannot be both activated to one (Constraint (2b)). Since multi-stream multiplexing is available in mm-wave backhauling, an SBS may route data streams through multiple separate relays to the destination. However, to avoid a device becoming extremely congested, the number of wireless data streams from one device should better be restricted by the number of RF chains available at the device, shown in Constraint (2c), where $f(N_{RF})$ is a function of the number of available RF chains. For example, the number of streams may be restricted to be no more than twice the number of RF chains if there are not many requested flows in the system, where $f\left(N_{RF}\right)=2N_{RF}$. However, if the link between $b_{i}$ and $b_{j}$ is connected by fibre, it is excluded from Constraint (2c).
- Flow constraint: For each flow, the outgoing data from the source and the incoming data to the destination must be larger than zero (Constraint (2d)). Meanwhile, there should be no incoming data to the source or outgoing data from the destination for every flow (Constraint (2e)). If full duplex is adopted, a relay may receive and transfer data simultaneously. For an SBS that is neither the source nor the destination of a specific flow, the incoming amount of data to the SBS and the outgoing amount of data from the SBS should be equal, which is depicted in Constraint (2f). Constraint (2g) shows that for any two SBSs, the total amount of data transmitted between them is limited by the corresponding link capacity.

**Figure 4:**
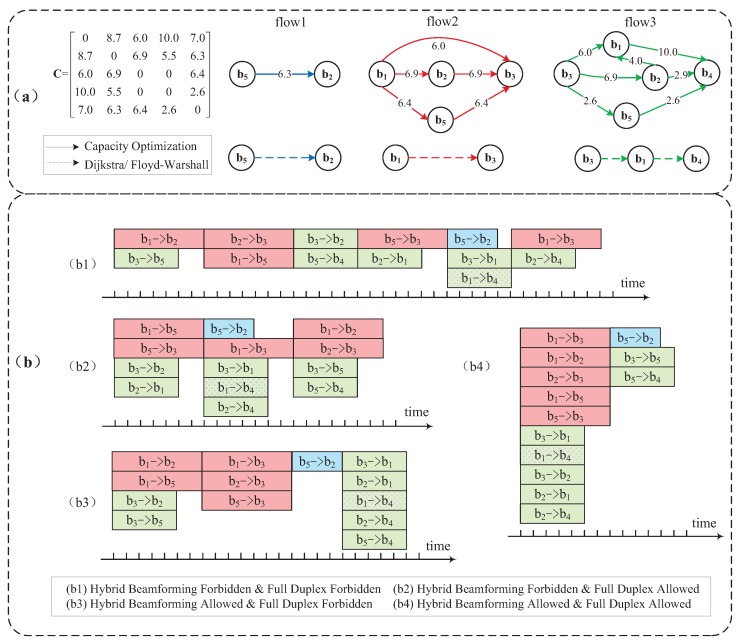
(**a**) Routing results of the shortest path algorithm and the capacity optimization method in the mm-wave backhaul network; and (**b**) the scheduling scheme in four scenarios based on the optimization routing results in (**a**) in a mm-wave backhaul network.

The above optimization problem is a mixed integer linear programming (MILP) problem, which could be solved by toolboxes, such as YALMIP [23]. Combining new physical layer techniques into path selection, the top part of Figure 4a shows an example of the routing result for three required flows, namely, $b_{5}\to b_{2}$, $b_{1}\to b_{3}$ and $b_{3}\to b_{4}$, in a five-SBS backhaul system using the above method. The matrix C stores the channel capacity (in units of Gbps) between each pair of devices. For a specific layout of SBSs, matrix C could be obtained from rate estimation through channel measurement. The setting of C for the example is shown in Figure 4a. The row index and column index refer to the SBS indices of the transmitter and the receiver, respectively. For example, the element in the first row and the second column of C shows that the capacity of the backhaul link from $b_{1}$ to $b_{2}$ is 8.7 Gbps. We assume that $b_{3}$ and $b_{4}$ are totally blocked, while there is an alternative link between $b_{4}$ and $b_{5}$ with higher loss. The wired link between $b_{1}$ and $b_{4}$ holds larger capacity. We assume that the available RF chains at each SBS in this example are three, and $f\left(N_{RF}\right)=N_{RF}=3$ due to the quite limited number of requested flows. It could be observed that the original Flow 2 and Flow 3 are separated into multiple new flows due to the availability of multi-stream beamforming. The number of incoming/outgoing flows at each SBS is restricted by $N_{RF}$, except that the number of flows sending from $b_{1}$ exceeds three, which is because the wired link from $b_{1}$ to $b_{4}$ is excluded from Constraint (2c). With the optimization goal of maximizing overall capacity, the rate for each new flow is re-allocated according to the full duplex constraint, *i.e.*, the total incoming flow rate and the outgoing rate at each SBS (neither source nor destination) are the same, as well as the capacity constraint, *i.e.*, the total rate of each link is lower than the link capacity. Compared to the conventional routing of Dijkstra/Floyd–Warshall shown in the bottom part of Figure 4a, the proposed routing scheme can fully exploit the potentials of mm-wave channels, leading to an improved backhaul performance.

### 5.3. Scheduling

After transmission path selection, the scheduling process is necessary to decide the transmission sequence of every hop in each requested flow. In conventional scheduling, links that can coexist are selected to transmit simultaneously. By considering new physical layer techniques in the mm-wave backhaul, one device may act as both the transmitter and the receiver of a single link or multiple links, and multiple hops in the same route may transmit together, which bring changes and challenges in scheduling.

#### 5.3.1. Optimization Model

Similar to routing, STDMA scheduling can be modelled by an integer optimization programming problem.

Minimizing transmission time: The whole scheduling is divided into several consecutive and non-overlapping time periods, defined as phases. Assume that there are *T* phases, and multiple links can transmit simultaneously in each phase. The maximum transmission time of the *t*-th phase is defined by its phase time, denoted as $\delta_{t}$, where 1≤t≤T. The goal of scheduling is to accomplish all of the transmissions with the minimum time; namely, to achieve:

(3) $$\min\sum_{t}\delta_{t}$$

System constraints: Before scheduling, every route should be separated into several single-stream transmissions from the source to the destination, each of which is called a new flow. All new flows are renumbered starting from one. A binary variable $h_{m,n}^{t}$ is defined to indicate that the *n*-th hop of new flow *m* is activated to send data in the *t*-th phase when it is assigned a value of one, and zero otherwise. The constraints of scheduling are shown as follows.

(4a) $$\begin{array}{c} \sum_{t}h_{m,n}^{t}=1\;\;\;\;\;\forall \;m,\;\;n \end{array}$$

(4b) $$\begin{array}{c} \sum_{m}\sum_{n}h_{m,n}^{t}\leq N_{RF}\;\left\{\begin{array}{c} \mathrm{if}\;\left\{m,n\right\}\in T(b_{i})\;\left(b_{i}\in B\right),\;\forall \;t\\ \mathrm{if}\;\left\{m,n\right\}\in R(b_{i})\;\left(b_{i}\in B\right),\;\forall \;t \end{array}\right. \end{array}$$

(4c) $$\begin{array}{c} \;h_{m_{1},n_{1}}^{t}+h_{m_{2},n_{2}}^{t}\leq 1\;\;\;\;\;\forall \;t,\;\forall \;\left\{m_{1},\;\;n_{1},\;\;m_{2},\;\;n_{2}\right\}\in I \end{array}$$

(4d) $$\begin{array}{c} \sum_{t}h_{m,n}^{t}\delta_{t}\geq d_{m}/r_{m,n}\;\;\;\;\;\forall \;m,\;\;n \end{array}$$

(4e) $$\begin{array}{c} \sum_{t=1}^{t^{*}}h_{m,\hat{n}}^{t}\geq \sum_{t=1}^{t^{*}}h_{m,\tilde{n}}^{t}\;\;\;\;\;\forall \;m,\;\;\hat{n}<\tilde{n},\;\forall \;t^{*}\in \left[1,T\right] \end{array}$$

- Device and channel constraint: In order to reduce the overhead brought by beam training and switching, we assume that each hop of a new flow would only be transmitted once, as shown in Constraint (4a). In each phase, both the scheduled numbers of incoming links to an SBS and outgoing links from an SBS should not exceed the number of RF chains, while wired links are excluded from such constraints. This constraint could be written as (4b), where $T(b_{i})$ and $R(b_{i})$ stand for wireless links with SBS $b_{i}$ as the transmitter and the receiver, respectively. Constraint (4c) shows that if there exists severe interferences between two specific links, e.g., the links corresponding to the $n_{1}$-th hop of the $m_{1}$-th new flow and the $n_{2}$-th hop of the $m_{2}$-th new flow, both of the links should not be activated at the same time. Set I refers to interference set. If the SINR value of at least one link is below a certain threshold when two links transmit simultaneously, these two links become an element in set I.
- Traffic and time constraint: Constraint (4d) shows that the time demand for the *n*-th hop of the *m*-th new flow, calculated from the data demands ($d_{m}$) and transmission rate ($r_{m,n}$) achieved by routing, should be no larger than its scheduled transmission time. For a specific new flow, the $\tilde{n}$-th hop could not be scheduled ahead of the $\hat{n}$-th hop, for $\hat{n}<\tilde{n}$, due to the relaying sequencing, shown in Constraint (4e). The full duplex techniques may enable a group of consecutive hops of a new flow to be transmitted in the same phase, which is exempted from half-duplex-related constraints in conventional scheduling problems.

The above optimization problem is a mixed integer nonlinear programming (MINLP) problem, which, however, can be converted into an MILP problem through the reformulation-linearization technique (RLT) [24]. After conducting the optimized routing scheme, Figure 4b illustrates the STDMA scheduling scheme in four independent scenarios based on the path selection result in Figure 4a. There are altogether eight new flows, with each containing two to three hops. Suppose that the link from $b_{1}$ to $b_{3}$ may interfere with the one from $b_{5}$ to $b_{4}$, so that they cannot be scheduled concurrently. Therefore, In every scenario in Figure 4b, link $b_{1}\to b_{3}$ and link $b_{5}\to b_{4}$ are allocated into different time phases. The time demands for the original flows, coloured in blue, red and green, are 4, 7 and 5 μs, respectively. The wired link between $b_{1}$ and $b_{4}$, depicted by dotted rectangles, is excluded from the constraints of hybrid beamforming. For example, in (b4), as long as the number of concurrent wireless links transmitted from $b_{1}$ does not exceed the number of RF chains, wired link $b_{1}\to b_{4}$ is able to be allocated in the same time period. Thanks to full duplexing, consecutive hops in a new flow are able to be allocated into the same phase. For example, as for the new flow $b_{3}\to b_{2}\to b_{1}\to b_{4}$ in (b2) where full duplex is allowed, the first two hops, $b_{3}\to b_{2}$ and $b_{2}\to b_{1}$, are allocated into the first phase. The last hop $b_{1}\to b_{4}$ is transmitted in the following phase, which is not allowed to be allocated in the previous phase of the first two hops due to relay sequencing. It is undoubted that the backhaul system with the capability of the both two new physical layer techniques ((b4) in Figure 4b) greatly reduces the total required transmission time and attains the best throughput performance, the throughput of which exceeds three times that of the STDMA scheduling scheme without using any new physical layer techniques ((b1) in Figure 4b).

#### 5.3.2. Algorithm and Solution

Since the complexity may become prohibitively high when the numbers of new flows and phases are very large, the optimization model of scheduling is time-consuming in dense small cells with explosive data demands. Simple, but effective heuristic algorithms become necessary to solve the problem. As an example, we provide a brief introduction of a scheduling scheme, which borrows the idea from the greedy colouring method given in [25]. The algorithm is conducted in chronological order, *i.e.*, we select and allocate the links that could be transmitted together in the first phase and then in the second phase, and so on. We define the unscheduled set as U, containing all unscheduled hops in every new flow based on the result of transmission path selection. For example, the original requested Flow 2 in Figure 4a is divided into three new flows, and corresponding elements in U for these new flows could be depicted as $\left\{b_{1},b_{3}\right\}$, $\left\{\left\{b_{1},b_{2}\right\},\left\{b_{2},b_{3}\right\}\right\}$ and $\left\{\left\{b_{1},b_{5}\right\},\left\{b_{5},b_{3}\right\}\right\}$, respectively, with a time length of 7 μs for each hop. The ultimate goal of the algorithm is to allocate all hops in U within the minimum total transmission time. We renumber all new flows from one, and define the visited matrix by V, with binary element $v_{i,j}$ representing that the *j*-th hop of the *i*-th new flow is visited when it is one, and zero otherwise. $v_{i,j}$ is initialized as zero if the corresponding hop exists, and one otherwise. For example, we assume that the indices of the above three new flows corresponding to the original requested Flow 2 in Figure 4a are 2, 3 and 4, respectively. Since there is only one hop in new Flow 2, $v_{2,1}$ and $v_{2,2}$ are initialized as zero and one, respectively.

In the scheduling of each phase, higher priority is given to the hop that occupies the most transmission time slots. The hops in U are rearranged in non-increasing order according to their required transmission time, and we search and select the hop from the beginning element of U. If the hop is available to be allocated in the current phase, its corresponding element in V is set as one. If the hop is not qualified to be transmitted together with hops that have been already allocated in the current phase, e.g., due to the constraints of the numbers of RF chains or the interference limitations, the corresponding elements of this hop and its following hops in the same new flow in V are all set as one. This is because if a hop has not been transmitted, the following hops of the same new flow should not be assigned ahead of it. Scheduling for each phase continues until V becomes an all-one matrix, which means no available hop is left unvisited. Set U is renewed at the end of each phase by removing the allocated hops. Meanwhile, corresponding elements of these allocated hops in V are set as one permanently. The algorithm terminates when U becomes empty, *i.e.*, all requested flows have been scheduled. The scheduling result by this heuristic method for the above example contains two phases; all hops except $b_{5}\to b_{4}$ are allocated into the first phase, and $b_{5}\to b_{4}$ is transmitted in the second phase due to the interference restriction. The total transmission time of the heuristic algorithm result turns out to be the same as that in (b4) in Figure 4b, *i.e.*, the optimal result for this particular example.

## 6. Case Study and Performance Evaluation

**Figure 5:**
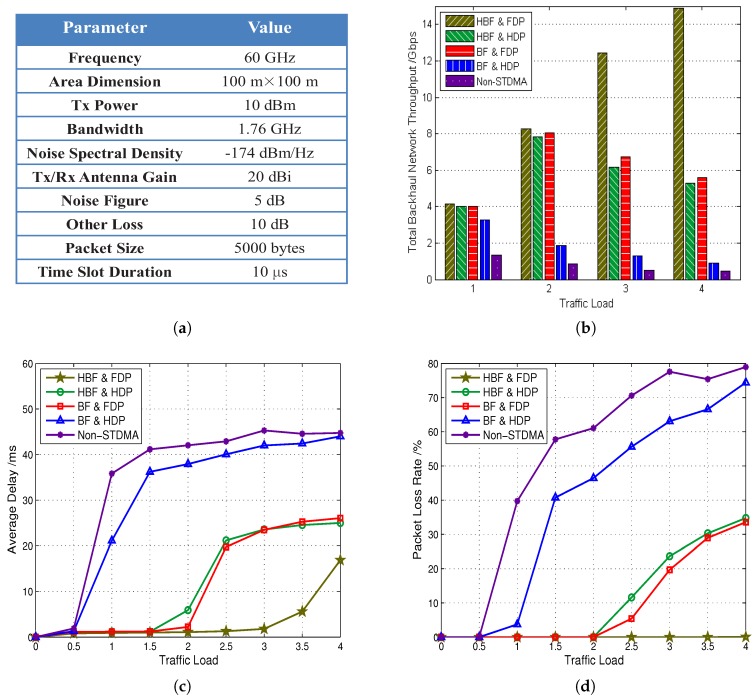
(**a**) Channel parameter settings and (**b**–**d**) the performance of the throughput, delay and packet loss rate of five schemes, respectively (HBF, BF, HDP and FDP refer to hybrid beamforming, analogue beamforming, half-duplex and full-duplex, respectively, while non-STDMA is the scheme that does not adopt spatial reuse at all).

We evaluate the performance of a simulated 60-GHz mm-wave backhaul network using the proposed framework, with the same network distribution as given in Section 3, *i.e.*, five SBSs are uniformly distributed in a 100 m × 100 m area. Fibre links, mm-wave links and two blockages are all considered to approach a realistic scenario. Two available RF chains are assumed at each SBS. Besides free space channel attenuation, other loss caused by rain attenuation, oxygen absorption and RF components in the mm-wave system is also considered in our simulation. We choose the capacity optimization to work out a routing scheme and then perform the STDMA scheduling using the heuristic method. We assume that the data demands arrive in units of packets based on the Poisson model [25] and is possible to be generated between any two SBSs. The total simulation time is $10^{6}$ time slots, each of which lasts 10 μs, and there are a small amount of required data initially. The MAC control overhead is assumed as two time slots in each frame. When a required datum has not been scheduled within 5000 time slots, it is discarded automatically. Detailed channel settings are listed in Figure 5a.

Figure 5b–d illustrate the performance of the throughput, delay and packet loss rate, respectively, achieved by the five schemes, where HBF, BF, HDP and FDP refer to hybrid beamforming, analogue beamforming, half-duplex and full-duplex, respectively, while non-STDMA is the scheme that does not adopt spatial reuse at all. The *x*-axis is the normalized traffic load. For example, a traffic load of two represents that the coming traffic requires a sum rate at every SBS in the backhaul system to be twice as the average capacity of a single link.

From Figure 5, we can observe that the performance of five schemes falls into three categories: (I) the proposed STDMA scheme supporting both new physical layer techniques (HBF and FDP); (II) two STDMA schemes adopting one new physical layer technique (HBF and HDP and BF and FDP); (III) two conventional schemes without new physical layer techniques or even without spatial reuse (BF and HDP and non-STDMA).

As shown in Figure 5b, the throughput of the proposed scheme grows rapidly with the increase of traffic load and keeps the highest among all schemes, which is more than twice that achieved by all other schemes at a heavy traffic load of four. The throughput of the schemes in Category II starts dropping at a traffic load of three, and the improvement brought by full duplex is slightly more obvious than that brought by hybrid beamforming, which supports two RF chains at each SBS. Undoubtedly, without the supports of new physical layer techniques or even without spatial reuse, the schemes in Category III achieve the least backhaul network throughput gain.

In Figure 5c, the growing speeds of the average delay of schemes in Category II and Category III slow down at traffic loads of 2.5 and 1.5, respectively. We conduct further explorations of the delay performance at higher traffic loads and find that the delay of the proposed scheme increases slowly when the traffic load is five. The convergence point of each scheme shows that when the traffic load exceeds a certain level, the spatial reuse capability of the scheme could hardly handle the quickly emerging large amount of required flows, and the network simply discards more packets without adding much delay. Since the upper bound of delay is 50 ms, the schemes in Category III already approach the upper bound at light traffic loads, while the other three schemes converge to a delay, which is only half of the upper bound.

The trend of the curves of the packet loss rate performance manifested in Figure 5d is similar to the delay performance. It should be noticed that the proposed scheme achieves almost zero packet loss even at a heavy traffic load of four, while more than 70% and 30% of packets have been discarded for the schemes in Category III and Category II, respectively.

In order to further explore the potential of the proposed framework, we enlarge the backhaul network size to contain six small cells and conduct throughput, delay and packet loss rate comparisons with the five-SBS network in scenarios where one, two and three wireless backhaul links are blocked. The traffic load is set as three, and the simulation results are listed in Table 2.

It could be observed that the throughput gain increases while the average delay and packet loss rate decrease in larger networks with fewer blockages. The proposed scheme outperforms all other schemes in every single occasion and shows more benefits in scenarios of larger numbers of blockages. For example, in a six-SBS backhaul network, the throughput gain of the proposed method is slightly larger than that of the schemes in Category II when there exists one blockage, while becoming approximately twice that of the schemes in Category II in the three-blockage scenario. The benefits brought by full duplexing are slightly more obvious than those brought by hybrid beamforming when comparing the results of HBF and HDP and BF and FDP in all scenarios, which is the same as the above observation in Figure 5. It should be noticed that no packet loss is guaranteed by adopting the proposed framework in all scenarios in Table 2, which shows the superiority of the robustness of the proposed scheme in comparison with other schemes.

## 7. Conclusions

Thanks to numerical channel measurements and successful demos, mm-wave communication has been considered as a feasible and potential technology for the outdoor 5G system, which could provide much larger transmission rates than conventional lower frequency communications. This article has demonstrated a backhaul system that adopts mm-wave links to replace most of the fibre links in a conventional backhaul network. A design framework has been proposed by exploiting several newly-developed physical layer technologies of mm-wave communication. By modelling the routing and scheduling together and developing optimization techniques to jointly examine them, the proposed framework significantly enhances the total capacity of the mm-wave backhaul network. Extensive simulation results have confirmed the achievable benefits of the proposed design framework. Compared to most proposals for 5G backhaul, which still considers conventional physical layer techniques, such as point-to-point beamforming, the proposed routing and scheduling scheme, which integrates new mm-wave physical layer techniques, achieves much larger throughput, a shorter delay and a smaller packet loss rate. This article thus may help open a new research direction for designing the next-generation wireless backhaul. In addition, the standardization support, e.g., hybrid beamforming, has been incorporated as one of the essential techniques in the next generation of the mm-wave system in the IEEE 802.11ay standard, as well as cost reduction due to progress in mm-wave equipment, which may both help facilitate the practical implementation of our proposed framework in the 5G network.

## Figures and Tables

**Table 1:** A brief summary of mm-wave communication in several frequency bands.

Frequency Band	Path Loss		Oxygen	Rain Attenuation		Coverage with
	100 m	200 m	Absorption	5 mm/h	25 mm/h	<20% Outage
28 GHz (K${}_{a}$ band)	50.69$\bar{n}$	53.70$\bar{n}$	0.2 dB/km	0.9 dB/km	4.5 dB/km	200 m
38 GHz (Q band)	52.02$\bar{n}$	55.03$\bar{n}$	0.15 dB/km	1.3 dB/km	7 dB/km	200 m
60 GHz (V band)	54.00$\bar{n}$	57.01$\bar{n}$	16 dB/km	2.2 dB/km	10 dB/km	100 m
73 GHz (E band)	54.85$\bar{n}$	57.86$\bar{n}$	0.45 dB/km	3 dB/km	12 dB/km	200 m
**Frequency Band**	**Advantages**			**Disadvantages**		
28 GHz	Suffers the least path loss; Low oxygen absorption and rain attenuation.			Lightly licensed; The bandwidth is relatively small.		
38 GHz	Relatively less attenuation caused by oxygen absorption and rain.			Less research and applications done.		
60 GHz	Unlicensed bands; Large bandwidth to achieve multi-gigabit rate.			Peak point of oxygen absorption; Relatively large rain attenuation.		
73 GHz	Small effects of atmospheric absorption.			Large rain attenuation; Large path loss due to high frequency point.		

$\bar{n}$: the path loss coefficient.

**Table 2:** System performance in scenarios of different numbers of small cell BSs (SBSs) and blockages.

Scheme	5-SBS Backhaul Network			6-SBS Backhaul Network		
	1 Blockage	2 Blockages	3 Blockages	1 Blockage	2 Blockages	3 Blockages
**Backhaul Network Throughput**						
HBF and FDP	14.48 Gbps	12.43 Gbps	9.92 Gbps	15.34 Gbps	13.54 Gbps	11.86 Gbps
HBF and HDP	6.50 Gbps	6.21 Gbps	3.39 Gbps	13.25 Gbps	9.91 Gbps	6.11 Gbps
BF and FDP	10.36 Gbps	6.72 Gbps	3.92 Gbps	14.83 Gbps	12.32 Gbps	6.86 Gbps
BF and HDP	2.43 Gbps	1.27 Gbps	0.66 Gbps	3.74 Gbps	2.15 Gbps	0.90 Gbps
None-STDMA	0.98 Gbps	0.50 Gbps	0.37 Gbps	1.71 Gbps	0.75 Gbps	0.43 Gbps
**Average Delay**						
HBF and FDP	1.56 ms	1.80 ms	3.15 ms	1.05 ms	1.14 ms	1.52 ms
HBF and HDP	23.07 ms	23.55 ms	25.23 ms	15.59 ms	20.46 ms	24.23 ms
BF and FDP	20.67 ms	23.52 ms	25.64 ms	3.35 ms	10.57 ms	23.05 ms
BF and HDP	41.09 ms	41.98 ms	43.48 ms	30.64 ms	30.55 ms	31.14 ms
None-STDMA	43.25 ms	45.56 ms	43.80 ms	31.12 ms	30.91 ms	32.49 ms
**Packet Loss Rate**						
HBF and FDP	0%	0%	0%	0%	0%s	0%
HBF and HDP	25.61%	23.28%	33.10%	0.16%	8.09%	21.98%
BF and FDP	8.69%	19.72%	31.28%	0%	0%	19.54%
BF and HDP	56.46%	63.20%	71.25%	45.61%	57.92%	67.89%
None-STDMA	68.51%	77.40%	73.90%	53.22%	72.97%	76.38%
